# Models will only get us so far: planning for place of care and death

**DOI:** 10.1093/ageing/afab070

**Published:** 2021-04-20

**Authors:** Erica Borgstrom

**Affiliations:** The Open University – Department of Health and Social Care School of Health, Wellbeing and Social Care Milton Keynes, UK

**Keywords:** advance care planning, COVID-19, end-of-life care, older people

## Key points

- Models for advance care planning can help facilitate decision-making.
- For models to be effective, they require that people have appropriate skills, knowledge and resources.
- Advance care planning has the potential to consider more than just place of care and death.

Over several decades, there has been increasing attention given to how, as a society and as individuals, we can ensure that people age and die well. On the latter point, end-of-life care policy has focused on place of death as a mechanism for and measure of improving the quality of care [1]. Being cared for and dying in one’s usual place of residence, including a care home, has been presumed to be both desirable and beneficial for dying people, those who care for them and the bereaved [2]. Nevertheless, operationalising this ambition across all groups and geographical areas has been difficult, not least because not everyone has the same preference and/or access to resources [3]. Moreover, the assumptions about the importance of ‘dying at home’ have been challenged [4].

Amongst this backdrop of thinking about if and how the location of care and death may matter, in countries like the UK, where the death toll from the coronavirus pandemic (COVID-19) has been high and ever present, societally and professionally we have been further challenged to reappraise how care is provided. Considering this, in a recent issue West *et al*. present a rapid review of reviews to understand place of care and death for older people and how this can be applied to current and future contexts [5]. They ask two main questions: what influences someone’s decisions about place and what influences the processes and outcome of place.

Importantly, place of care and place of death can indeed be different places for the same individual as found by West *et al*. There may be multiple places of care, and a change of preferences, across the time someone is at the end of their life. Nevertheless, place of care and place of death are often conflated and viewed as static in the literature and indeed, this can also be seen in patient notes, audit documents or policy statements. Additionally, whilst home is often the presumed preference, there is considerable heterogeneity and care-givers may not be accurate proxies [6]. Through their review, West *et al*. highlight this variety and the importance of determining if preferences vary.

Consequently, they’ve developed a decision-making model to support care provision aligned with people’s preferences, which can be used both during the current pandemic and in other circumstances. Whilst their review was limited to research concerning older people, their model has general relevance when considering end-of-life care.

What underlies much of the literature considered by West *et al*., and indeed the model presented, is a logic of advance care planning and patient choice [7, 8]. At its core, this logic presumes that considering and discussing preferences—for example around preferred place of death—can enable patients, informal carers and care professionals to align care provision to the preference [9]. The decision-making model presented here suggests ways in which to support these elements with an emphasis on the information people may need, including information that is accessible, culturally appropriate and in a language the person can understand. The model also encourages considering the decision-making context, such as mental capacity and the network of people involved. Although they do not go as far as to suggest relational autonomy, there is an understanding that some of the decisions being made are not just about the older person. This is evident in their example of how advance care plans may need to be renegotiated and reconsidered as care options change during the COVID-19 pandemic, such as the impact of visitor restrictions. Others have also recommended thinking about joint discussions and plans to acknowledge the COVID-19 context [10, 11].

Advance care planning is an evolving field [12] and this may be a useful model for many for capturing a variety of aspects to be considered during the ongoing process of decision-making. However, like most models, it will not in itself do the work. It requires people being willing or able to articulate their preferences—and some may not wish to do this or may consider the future differently to how advance care planning orients time. As seen in other studies about shared decision-making and advance care planning, for it to be effective, those employing this model still require skills and judgement on how to communicate, how to weigh up different factors and manage potential tensions between these elements and/or between people. It takes a commitment to have conversations early, revisit decisions once they have been made and accept that they can be fluid, which has not always been done before [13]. Moreover, for the outcomes to be realised, there will need to be an ongoing effort to ensure the preferences and decisions can be enacted even if they change: assessing needs, providing supports, facilitating adaptation. Patient, families, carers and professionals therefore not only need access to information, but also a range of skills and resources that facilitate making preferences a reality.

If aligning place of care and death with the dying person’s preference is considered an outcome, the process of advance care planning can be a useful mechanism for determining and preparing for such preferences. However, place is not the only ‘outcome’ that matters to many people—they may be more concerned about how they are cared for, or who they can see, or how much control they feel they have. Advance care planning can consider a much wider remit than just location of care, and it may be the processes of discussion that is most beneficial for some. Importantly, decisions—in how they are made, enacted and experienced—are inherently relational, involving and impacting the dying person and also those who care about them and for them, including health and social care professionals. There is therefore further scope to consider what good outcomes look like for this wider network and how to support them all through the changing contexts of care during the end-of-life and the COVID-19 pandemic. Models can be useful to support conversations but they will only get us so far.
